# The Effect of Pectinase-Assisted Extraction on the Physicochemical and Biological Properties of Polysaccharides from *Aster scaber*

**DOI:** 10.3390/ijms19092839

**Published:** 2018-09-19

**Authors:** Young-Ran Song, Su-Kyung Sung, Eun-Ju Shin, Chang-Won Cho, Chun-Ji Han, Hee-Do Hong

**Affiliations:** 1 Korea Food Research Institute, Wanju-gun, Jeollabuk-do 55365, Korea; song.young-ran@kfri.re.kr (Y.-R.S.); sung.su-kyung@kfri.re.kr (S.-K.S.); shin.eun-ju@kfri.re.kr (E.-J.S.); cwcho@kfri.re.kr (C.-W.C.); 2 Medical College, Yanbian University, Yanji 133002, China; chjihan@126.com

**Keywords:** *Aster scaber*, polysaccharide, enzyme-assisted extraction, characterization, immunostimulatory activity

## Abstract

The edible and medicinal perennial herb *Aster scaber* is known to have anticancer, antioxidant, and immunomodulatory properties. However, the biological effects of its polysaccharides are not well understood. Here, we aimed to extract novel polysaccharides with enhanced biological properties from *Aster scaber* using enzyme-assisted methods. Amylase, cellulase, and pectinase were used to extract enzyme-assisted polysaccharide (ASEP)-A, ASEP-C, and ASEP-P, respectively. The yields, physicochemical properties, and immunostimulatory activities of the polysaccharides were investigated and compared with those of hot water extracted polysaccharide (ASWP). The highest yield (3.8%) was achieved for ASEP-P extracted using pectinase digestion. Fourier-transform infrared spectroscopy (FT-IR) and chemical composition analysis revealed that ASWP and three ASEPs were typical acidic heteropolysaccharides, mainly comprising rhamnose, arabinose, galactose, glucose, and galacturonic acid. Immunostimulatory activity assays on RAW264.7 macrophages showed ASEP-P to have the greatest immunostimulatory potential in terms of nitric oxide (NO) and cytokine productions and phagocytic activity. ASEP-P administration improved immune-enhancing effects in normal mice by improving the spleen index and splenic lymphocyte proliferation, and in immunosuppressed mice by modulating lymphocyte proliferation, natural killer (NK) cell activity, and leukocyte counts. The ASEP-P derived from pectinase hydrolysate of *Aster scaber* demonstrated efficacious immunostimulatory properties and has potential applications as an immune stimulator.

## 1. Introduction

Polysaccharides are important biological macromolecules and have attracted considerable attention due to their biocompatibility, low toxicity, unique physical properties, and specific therapeutic properties [1]. Many natural polysaccharides derived from plants possess proven biological properties, including antitumor, antioxidant, antibacterial, anti-inflammatory, and immunoregulatory activities [2,3,4,5]. Immunostimulation in particular is one of the most important actions of polysaccharides. Immunostimulatory polysaccharides can interact directly or indirectly on the immune system by inducing cellular/molecular events and strengthening innate and adaptive immune responses [6].

*Aster scaber* Thunb. (syn. *Doellingeria scabra* Thunb.), an edible perennial plant of the family Asteraceae, is widely cultivated in Eurasia, including China, Japan, and Korea [7]. It has long been used as a traditional medicine for various diseases and conditions such as bruises, dizziness, headaches, and snakebites [8]. *Aster scaber* has been shown to possess various pharmacological properties such as antioxidant, anticancer, antiviral, antihepatotoxic, neuroprotective, and immunomodulatory activities [8,9,10,11]. These effects have been attributed to the low-molecular-weight phytochemicals present in the plant, including triterpene glycosides, monoterpenes, saponins, polyphenolics, and volatile compounds [7,11]. However, little is known about the biological effects of polysaccharides isolated from *Aster scaber*, despite its high carbohydrate content of approximately 70% of dry weight of *Aster scaber* [12]. Recently, we reported that the polysaccharide fraction isolated from *Aster scaber* using hot water extraction enhanced the secretion of nitric oxide (NO) and cytokines such as interleukin (IL)-6, and tumor necrosis factor (TNF)-α in macrophages, and demonstrated anti-complement activity [13].

Many researchers have studied the extraction, purification, structure elucidation, and pharmacological activities of plant-derived polysaccharides [6,14,15,16,17]. The extraction techniques significantly affect the yield, physicochemical properties, and biological activities of polysaccharides [18]. Research on the extraction yield of polysaccharides is garnering increased attention due to improved extraction techniques. However, the impacts of the extraction process on the structure and biological characteristics of the polysaccharides tend to be neglected [19]. Hot water extraction, heat reflux, maceration, and acidic hydrolysis are commonly used to extract polysaccharides from natural resources [20]. However, they require large volumes of solvents, high temperature, long extraction times, or expensive equipment. Moreover, the harsh conditions can also damage the structure of the polysaccharides, thereby affecting the biological activity. In recent years, several new and innovative methods (e.g., enzyme-assisted extraction, microwave-assisted extraction, and ultrasound-assisted extraction) have extracted polysaccharides from natural resources [21]. Enzyme-assisted extraction is considered a mild, efficient, and environmentally friendly method, with several advantages, such as low energy consumption and easy operation standards [3]. The assistance of enzymes, which have been employed frequently as biocatalysts to obtain target compounds from various plants, can improve the extraction efficiency, yield, and/or biological activities of polysaccharides [22]. Many studies have demonstrated enzyme-assisted extraction to be an effective method to obtain polysaccharides with enhanced functionality from plant sources [4,14,16,21,22].

In this study, we aimed to use an enzyme-assisted method to extract novel polysaccharides derived from *Aster scaber* with enhanced biological properties. The immunostimulatory activity and physicochemical properties of polysaccharides were compared with those produced by hot water extraction. Furthermore, we evaluated the immune-enhancing properties of the polysaccharide extracted by the enzyme treatment with the highest immunostimulatory activity in normal and cyclophosphamide (Cy)-induced immunosuppressed mice.

## 2. Results and Discussion

### 2.1. Preparation of the Extracts

Generally, many plant polysaccharides are obtained in soluble form using hot water extraction. However, enzyme treatment has recently become a preferred technique to more efficiently extract bioactive polysaccharides with different structures and molecular weights. For enzyme-assisted extraction, enzymes such as pectinase, cellulase, α-amylase, and proteases have been used most frequently [4,14,15]. In our study, three polysaccharide fractions (ASEP-A, ASEP-C, and ASEP-P) were obtained from *Aster scaber* using the enzyme-assisted method and the following three enzymes: α-amylase, cellulase, and pectinase, respectively, while a polysaccharide fraction (ASWP) obtained with hot water extraction was used for comparison. As shown in Table 1, the yields of the four fractions were determined as ASEP-C (3.1%) < ASWP (3.3%) < ASEP-A (3.4%) < ASEP-P (3.8%), clearly indicating that the fraction obtained by pectinase digestion had the highest yield. The results indicate that the enzymatic extraction efficiency differed among the three enzymes. Meanwhile, these polysaccharide yields were lower than expected considering the high carbohydrate content of the dried *Aster scaber* [12].

### 2.2. Comparison of Physicochemical Properties of ASWP and Three ASEPs

ASWP and three ASEPs were characterized chemically and structurally. As shown in Table 1, the carbohydrate contents of all samples were >97% on the basis of the neutral sugar and uronic acid levels. ASEP-A and ASEP-P showed significantly higher neutral sugar and lower uronic acid contents than those in ASWP and ASEP-C. The protein content was significantly higher in ASWP than in the fractions by the three enzymatic extractions. In addition, ASEP-P showed higher levels of 2-keto-3-deoxy-mannooctanoic acid (KDO)-like materials than the others did. KDO is a unique component of rhamnogalacturonan-II (RG-II) among many plant polysaccharides [16], indicating that the RG-II polysaccharide is more abundant in ASEP-P by pectinase-assisted extraction. Here, monosaccharide compositions of four polysaccharide fractions were also determined (Table 1 and Figure S1), in which seven neutral sugars and two uronic acids were detected. The results indicated that rhamnose, arabinose, galactose, glucose, and galacturonic acid were the main monosaccharides of four polysaccharides. However, the molar ratios of these monosaccharide components of ASWP, ASEP-A, ASEP-C, and ASEP-P were significantly different: 0.44:1.00:0.44:1.00:3.15, 1.59:0.75:1.14:1.00:1.50, 0.62:1.25:0.67:1.00:2.63, and 2.08:1.11:1.74:1.00:2.03, respectively. Galacturonic acid appeared to be the most abundant of all polysaccharides. Compared with ASWP, the levels of rhamnose and galactose were significantly higher in ASEP-A and ASEP-P. Meanwhile, ASEP-C showed the highest level of arabinose. Plant cell walls are complex and dynamic structures formed by a heterogeneous mixture of cellulose, hemicelluloses, pectins, and glycoprotein [16]. As structural heteropolysaccharides are present in the cell wall, pectic polysaccharides contain a high proportion of galacturonic acid, which is mainly substituted by rhamnose residues with side chains consisting of neutral sugars, mainly rhamnose, arabinose, and galactose [6]. Thus, our results suggested that pectic polysaccharides were present as the main polysaccharides of *Aster scaber*, and that monosaccharide compositions of the polysaccharides can be affected by different enzymes and extraction methods. In addition, one possible explanation would be that ASEP-P had a higher content of KDO-like materials, rhamnose, and galactose, which might indicate the more abundant RG-II polysaccharide in ASEP-P.

**Figure 1:**
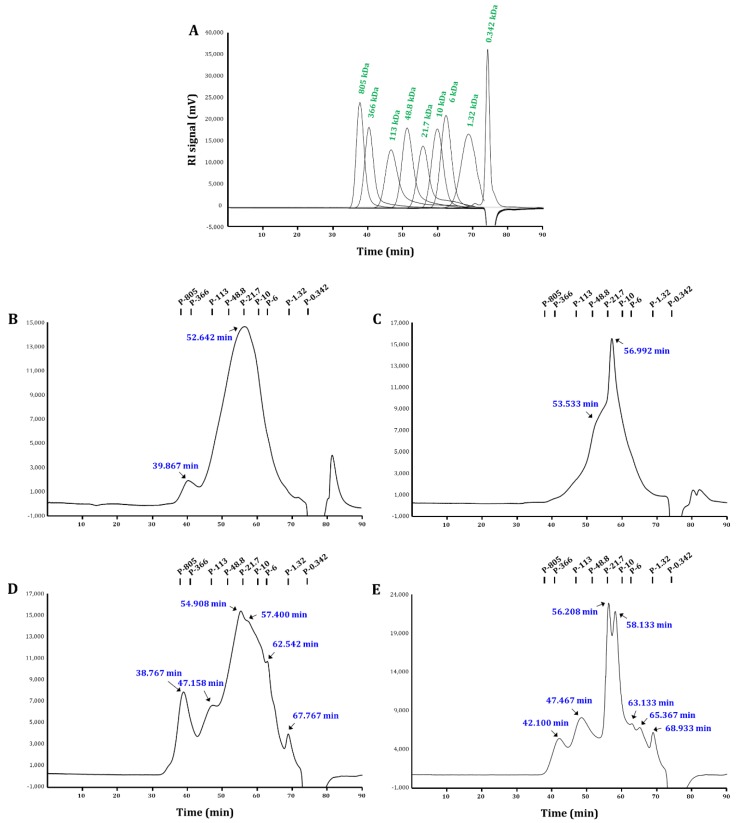
High-performance size-exclusion refractive index (HPSEC-RI) chromatograms of (**A**) pullulan standards, (**B**) ASWP, (**C**) ASEP-A, (**D**) ASEP-C, and (**E**) ASEP-P. ASWP was obtained from *Aster scaber* using hot water extraction; ASEP-A, ASEP-C, and ASEP-P were obtained from *Aster scaber* using α-amylase, cellulase, and pectinase, respectively, in an enzyme-assisted extraction.

Next, the molecular weight distributions of the polysaccharide fractions were confirmed by high-performance size-exclusion chromatography (HPSEC) relative to pullulan standards (Figure 1). As shown in the refractive index (RI) chromatograms, different extraction methods had significant effects on the molecular weight profiles. Here, all four fractions possessed several peaks. However, their molecular weight patterns were apparently different. ASWP had a large major peak at the elution times of 44–69 min, with the average molecular weight value of 34.9 × 10^3^ g/mol, and a small peak at 458.6 × 10^3^ g/mol. ASEP-A (extracted using α-amylase) showed two distinct groups, with average molecular weights of 29.1 and 14.5 × 10^3^ g/mol. ASEP-C and ASEP-P (extracted using cellulase and pectinase, respectively) showed more peaks of different molecular weight values throughout the retention time (572.5, 105.4, 22.1, 13.4, 4.7, and 1.6 × 10^3^ g/mol in ASEP-C; 292.3, 99.0, 17.0, 11.5, 4.2, 2.7, and 1.3 × 10^3^ g/mol in ASEP-P), having some oligomers with molecular weights of <5 × 10^3^ g/mol. Generally, pectic polysaccharides can have a large range of molecular weights, up to even ~1600 × 10^3^ g/mol, and their molecular weight distribution, structure, and size can impact the behavior of the polysaccharides [2,6]. In this study, polysaccharides from *Aster scaber* showed various molecular weight values, ranging between 572.5 and 1.3 × 10^3^ g/mol. This is the first report to determine the molecular weight of *Aster scaber* polysaccharides. Furthermore, it was confirmed that molecular weight patterns of polysaccharides were markedly affected by different extraction methods; in particular, cellulase and pectinase digestions were attributed to obtain more complex polysaccharides from *Aster scaber*.

**Figure 2:**
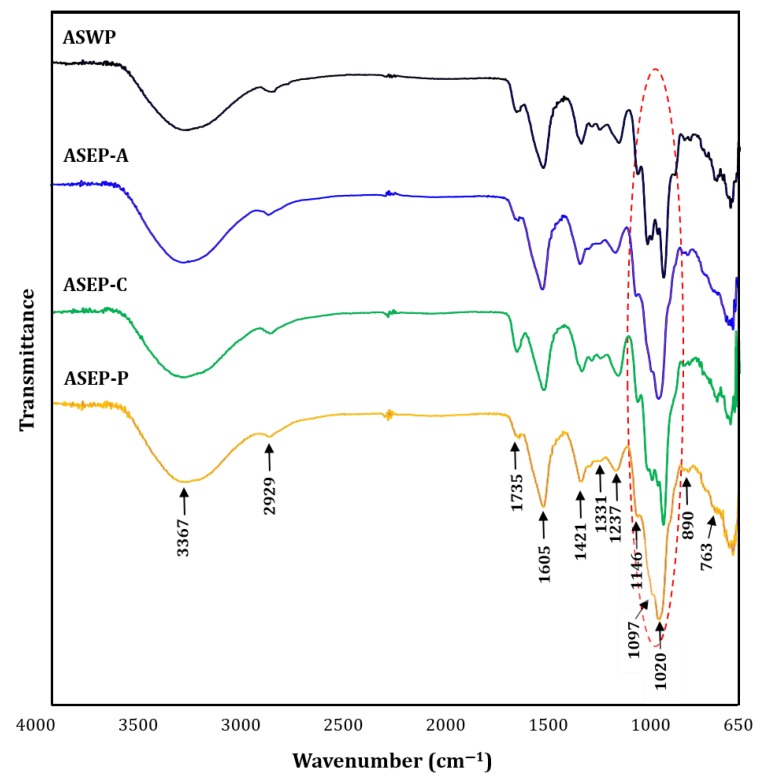
Fourier-transform infrared spectroscopy (FT-IR) spectra of ASWP and three ASEPs. ASWP was obtained from *Aster scaber* using hot water extraction; ASEP-A, ASEP-C, and ASEP-P were obtained from *Aster scaber* using α-amylase, cellulase, and pectinase, respectively, in an enzyme-assisted extraction. The red dashed circle indicates the stretching peaks in the region of 1200–1000 cm^−1^.

The infrared spectra clearly revealed characteristic absorption peaks of the polysaccharides and can thus be used for the identification of various functional groups of polysaccharides [23]. Figure 2 shows the Fourier-transform infrared spectroscopy (FT-IR) spectra of ASWP and three ASEPs. The four fractions all displayed similar absorption patterns, except for the patterns of stretching peaks ranging from 1200–1000 cm^−1^. Specifically, the broad and intense band at approximately 3367 cm^−1^ and the weak band at 2929 cm^−1^ of the four fractions were ascribed to the O–H stretching vibration and the C–H stretching vibration of CH_2_, respectively [5]. The absorption peak at 1605 cm^−1^ might be due to bound water. The absorption bands at around 1735 and 1421 cm^−1^ were attributed to the presence of carboxyl and carbonyl groups, respectively, indicating the characteristic of acidic polysaccharides, which was in accordance with the results of the chemical compositions [24]. In addition, the two peaks at 1331 and 1237 cm^−1^ might also be due to the presence of sulfate ester groups [17], but this needs to be confirmed further. Simultaneously, the strong absorptions in the region of 1200–1000 cm^−1^ were derived from the ring vibrations overlapping with the stretching vibrations of the C–OH side groups and the C–O–C glycosidic bond vibrations, indicating the four polysaccharides contained pyranose monomers [23]. However, the polysaccharide samples showed different patterns for the stretching peaks in the region of 1200–1000 cm^−1^. Generally, polysaccharides show high absorptions in the sugar region of 1200–950 cm^−1^, and in particular absorption pattern differences in the region can be used to discriminate sugar components of polysaccharides [25]. Thus, in this study, these differences in the four polysaccharide fractions might be attributed to their different physicochemical properties, which is consistent with the result of the monosaccharide analysis. In addition, the absorption peaks at 890 and 763 cm^−1^ were evidence of the presence of a β-configuration and d-glucopyranose ring, respectively [18]. Overall, our results demonstrated that ASWP, ASEP-A, ASEP-C, and ASEP-P showed the typical absorption peaks of polysaccharides, having a similar backbone and chemical groups, but that sugar compositions of *Aster scaber* polysaccharides can be affected by different extraction methods.

### 2.3. Comparison of Macrophage Immunostimulatory Activity of ASWP and Three ASEPs

**Figure 3:**
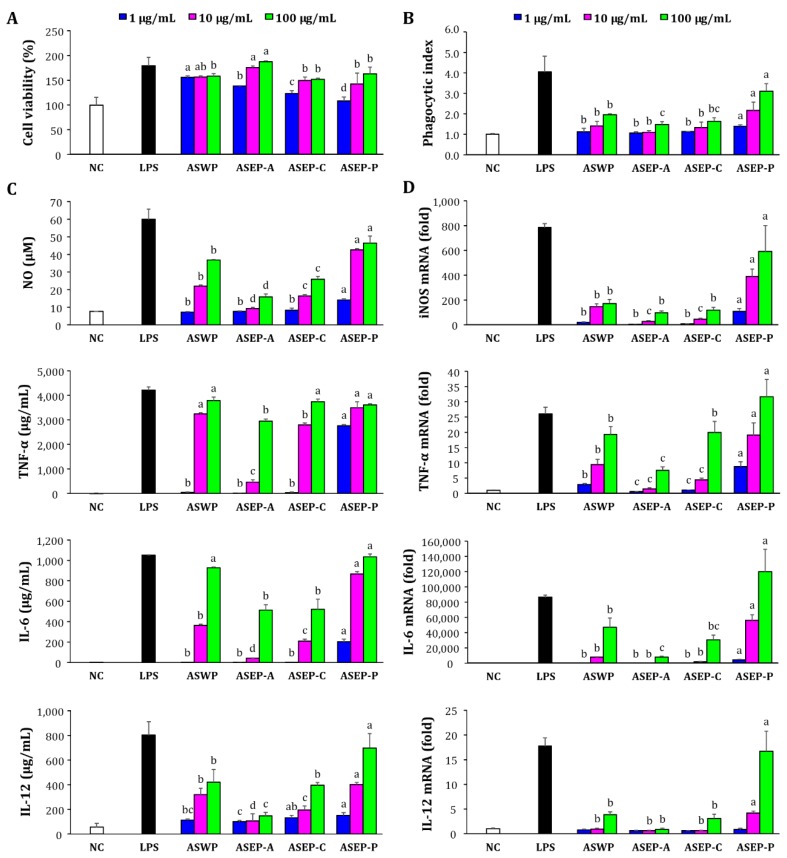
Effects of ASWP and three ASEPs on cell viability; phagocytic activity; and the production of nitric oxide (NO), tumor necrosis factor (TNF)-α, interleukin (IL)-6, and IL-12 in RAW264.7 macrophages. ASWP was obtained from *Aster scaber* using hot water extraction and ASEP-A, ASEP-C, and ASEP-P were obtained from *Aster scaber* using α-amylase, cellulase, and pectinase, respectively, in an enzyme-assisted extraction. RAW264.7 macrophages were stimulated with the polysaccharide samples at 1, 10, or 100 μg/mL. LPS (1 μg/mL) was used as the positive control (PC). (**A**) Cell viability was determined by the cell counting kit-8 (CCK-8) assay; (**B**) Phagocytic activity was determined using a phagocytosis assay; (**C**) NO levels in the culture media were determined by measuring nitrite accumulation, and the secretion levels of TNF-α, IL-6, and IL-12 were measured by ELISA. (**D**) mRNA expression levels of iNOS, TNF-α, IL-6, and IL-12 in macrophages were quantified by qRT-PCR analysis. Lowercase letters (a–c) indicate significant differences (*p* < 0.05) among four different polysaccharide samples at the same concentration.

Macrophages, which are widely distributed throughout the body, participate in both specific and non-specific immune reactions [23]. Macrophages protect the host by triggering the phagocytosis of macrophages and secreting macrophage-derived bioactive molecules such as IL-1β, IL-6, IL-12, TNF-α, and NO, which regulate the activity of other cells [5]. In this study, the immunostimulatory effects of the four polysaccharide fractions, ASWP, ASEP-A, ASEP-C, and ASEP-P, were assessed using RAW267.4 macrophages (Figure 3). As shown in Figure 3A, the cell viability was greater than 100% at all doses tested (1–100 µg/mL), indicating that the polysaccharides were not cytotoxic to RAW267.4 cells. Phagocytosis is one of the most distinguished features of macrophage activation [17]. The effects of four samples on the phagocytic capacity of macrophages were also evaluated. As shown in Figure 3B, the phagocytosis index increased with the treatments using all four samples. Compared with ASWP, higher stimulating effects on the macrophages were found with ASEP-P at all treatment concentrations. Meanwhile, ASEP-A resulted in the lowest stimulant activity on the phagocytic activity of macrophages.

In addition, the four fractions were tested for their roles in the regulation of macrophage-stimulating factors such as NO, TNF-α, IL-6, and IL-12, which increase both cell-mediated and immune responses [26]. The stimulation of four polysaccharide samples significantly promoted the secretion of NO, TNF-α, IL-6, and IL-12 in RAW264.7 cells compared with the untreated cells, which occurred in a dose-dependent manner, indicating that all samples could induce the production of these cytokines in the macrophages (Figure 3C). Among them, ASEP-P could dramatically promote the secretion of these cytokines over the concentration ranges tested. In fact, the level stimulated with ASEP-P at 100 µg/mL was comparable to that by the treatment of the positive control LPS. In addition, qRT-PCR analysis was carried out to confirm whether cytokine secretion from RAW264.7 cells of the polysaccharides was due to the increased expression of the relevant gene associated with NO and cytokine productions. As shown in Figure 3D, all four polysaccharides could upregulate the mRNA expression of iNOS, TNF-α, IL-6, and IL-12 in a dose-dependent manner, consistent with the above results of NO or cytokine secretion. Of note, ASEP-P was the most effective, in which ASEP-P induced a considerably high mRNA expression of iNOS in RAW264.7 cells, suggesting that the increased NO secretion from the cells might be attributed to the enhanced mRNA expression of iNOS via activation of the macrophages by ASEP-P. Moreover, the expression levels of TNF-α, IL-6, and IL-12 at 100 µg/mL ASEP-P exceeded that of the LPS group (*p* < 0.05). Taken together, these results suggested that macrophages can be activated using *Aster scaber* polysaccharides. Evidently, ASEP-P extracted by pectinase assistance was more effective in enhancing effects than the other fractions were, including ASWP. This suggested that pectinase digestion is a useful method to extract immunostimulatory polysaccharides from *Aster scaber*. In contrast, α-amylase and cellulase might not be suitable for extracting immunostimulatory polysaccharides from *Aster scaber.*

### 2.4. Immuno-Enhancement Effects of ASEP-P In Vivo

#### 2.4.1. Effects on Immune Organ Indices and Splenic Lymphocyte Proliferation in Normal Mice

As the most potent immunostimulatory polysaccharide of *Aster scaber*, the immune-enhancing effect of ASEP-P was further evaluated in both normal and immunosuppressed mouse models. In normal mice, the effects of ASEP-P administered at doses of 100 and 200 mg/kg (body weight) determined immune organ indices and splenic cell proliferation (Table 2). The body mass gain in the treated groups differed significantly from that in the control group (*p* > 0.05), indicating that there was no adverse effect on the mice. However, the spleen index significantly increased following treatment, with ASEP-P at 200 mg/kg compared with that in the control group. Furthermore, the thymus index tended to increase in the ASEP-P treated groups compared with that in the control group, but the differences were not statistically significant. The spleen is the largest lymphoid organ, containing various immune cells, including T- and B-lymphocytes, macrophages, and dendritic cells, among which T-lymphocytes are associated with adaptive or cell-mediated immune responses and B lymphocytes are the key elements involved in humoral immune responses [27]. Generally, immunostimulants can enhance the weight of immune organs and the proliferation of immune cells [28]. Compared to the control group, T- and B-lymphocyte proliferation was higher by ASEP-P (100 and 200 mg/kg) treatment. The results suggested that ASEP-P can stimulate the development of immune organs and cells in normal mice and thus might enhance the immune system.

#### 2.4.2. Effects on Splenic Lymphocyte Proliferation, Natural Killer Cell Activity, and White Blood Cell Count in Cyclophosphamide-Treated Mice

**Figure 4:**
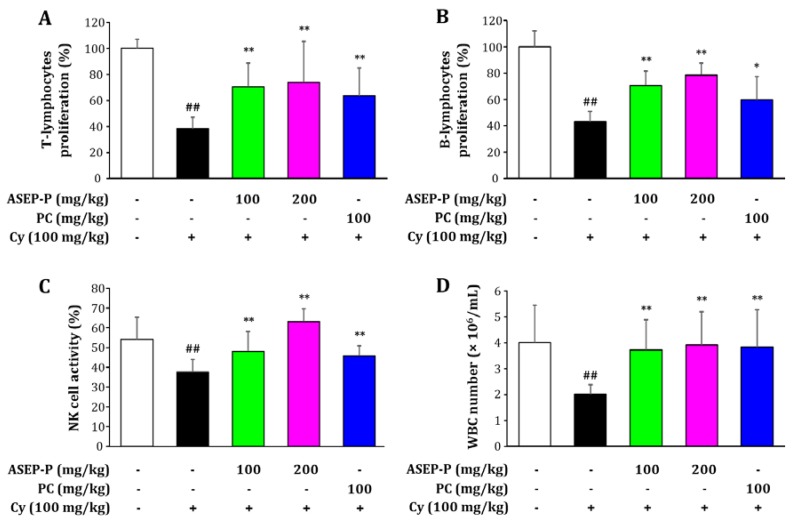
Effects of ASEP-P on (**A**) T-lymphocyte proliferation, (**B**) B-lymphocyte proliferation, (**C**) natural killer (NK) cell activity, and (**D**) white blood cell (WBC) counts in cyclophosphamide (Cy)-induced immunosuppressed mice. The mice were pre-treated with Cy (100 mg/kg for 3 days) for immunosuppression and then orally administered ASEP-P (100 or 200 mg/kg for 14 days). CVT-E002™ (100 mg/kg) was used as a PC. The data are expressed as the means ± standard deviation (*n* = 10); ^##^ *p* < 0.01, compared with the vehicle-treated control group; * *p* < 0.05 and ** *p* < 0.01 compared with the Cy-treated model group.

Although Cy is an important chemotherapeutic drug used for tumor treatments, it has adverse effects on the immune system, such as leukopenia, myelosuppression, and immunosuppression [29]. In this study, compared to the normal group, the Cy-treated model group showed a markedly decreased proliferation of T- and B-lymphocytes, natural killer (NK) cell activity, and white blood cell (WBC) count, indicating a successful induction of immunosuppression (Figure 4). However, the treatment with ASEP-P exerted obvious chemoprotective effects on Cy-induced immunosuppression. The administration of ASEP-P at 100 and 200 mg/kg significantly improved spleen lymphocyte proliferation induced by Cy treatment, suggesting a role for ASEP-P in the activation of T- and B-lymphocytes. The NK cell activity and WBC count in the groups treated with ASEP-P (100 and 200 mg/kg) were also significantly higher than those in the Cy-treated group. In addition, CVT-E002™, a positive control (PC), increased the lymphocyte proliferation, NK-cell activity, and WBC count. As a type of lymphocyte, NK cells exhibit a nonspecific killing activity targeting foreign and abnormal cells in the early phases of immune defense [30]. Leukocytes, a part of the body’s immune system, also provide defense against infectious diseases and foreign invaders, and excessive Cy administration is known to decrease the number of leukocytes and thus induce leukopenia [29]. Here, ASEP-P could effectively prevent the Cy-induced pathological changes, simultaneously increasing lymphocyte proliferation, NK cell activity, and the number of WBC in Cy-treated immunosuppressed mice. Taken together, these results also support the conclusion that ASEP-P can boost host immunity, irrespective of the host being in a normal or weakened state.

## 3. Materials and Methods

### 3.1. Materials

Samples of *Aster scaber* Thunb. was cultured and collected from Goheung (Jeollanam-do, Korea) in 2016, when it was dried and used for the experiments. Three commercial enzymes were obtained from Vision Corporation (Seongnam, Gyonggi, Korea) and used for enzymatic-assisted extraction of polysaccharides from *Aster scaber*: (1) thermostable α-amylase (EC 3.2.1.1) from *Bacillus licheniformis* (Spezyme^®^; optimal temperature, 35–95 °C; optimal pH, 4.5–6.0); (2) cellulase (EC 3.2.1.4) from *Trichoderma reesei* (Rohament^®^; optimal temperature, 50–60 °C; optimal pH 4.5–5.0); (3) and pectinase (EC 3.2.1.15) from *Aspergillus niger* (Plantase MAX^®^; optimal temperature, 10–55 °C; optimal pH, 3.0–5.0; activities: polygalacturonase and pectinesterase).

### 3.2. Extraction of Polysaccharides from Aster scaber

#### 3.2.1. Polysaccharides Obtained Using Enzyme-Assisted Extraction

For the preparation of ASEP, the dried *Aster scaber* (500 g) was crushed, combined with distilled water at a 1:10 (*w*/*v* raw material) ratio, and then extracted in a 10 L batch reactor using three different commercial enzymes. The mixture was adjusted to an initial optimal pH of 4.5–5.0, and then, each enzyme (1%, *v*/*w* raw material) was added. The enzyme-assisted extraction was performed at 90 °C for 24 h with α-amylase or at 50 °C for 24 h with cellulase and pectinase. The enzyme hydrolysate was heated at 100 °C for 20 min to inactivate the enzymes and centrifuged at 6500× *g* for 20 min at 4 °C to remove insoluble material. The supernatant was precipitated by the addition of three volumes of cold ethanol at −20 °C overnight to obtain crude polysaccharides. The precipitate was dialyzed using a Spectra/Por^®^ membrane (molecular weight cutoff, 3000–6000 Da; Spectrum Laboratories Inc., Rancho Dominguez, CA, USA). The high-molecular-weight fraction was finally lyophilized to yield ASEP; specifically, ASEP-A by α-amylase, ASEP-C by cellulase, or ASEP-P by pectinase.

#### 3.2.2. Polysaccharides Obtained Using Hot Water Extraction

ASWP was prepared from *Aster scaber* in the same manner as ASEP, except that instead of pH adjustment and enzyme treatment, the sludge was extracted twice (4 h each) at 100 °C.

### 3.3. Physicochemical Properties of Polysaccharides

#### 3.3.1. Chemical Component Analysis

The neutral sugar and uronic acid contents were determined by both the phenol–sulfuric acid method with glucose as the standard [31], and by the carbazole–sulfuric acid method with d-galacturonic acid as the standard [32]. The protein content was assessed using the Bradford method with bovine serum albumin as the standard [33], and the content of KDO-like materials was assessed by the modified thiobarbituric acid-positive method using KDO [34].

#### 3.3.2. Monosaccharide Composition Analysis

Monosaccharide composition was analyzed using high-performance anion exchange chromatography and coupled with pulsed amperometric detection (HPAEC-PAD; ICS-5000, Dionex Co., Sunnyvale, CA, USA) after hydrolysis with 2 M trifluoroacetic acid at 100 °C for 4 h. The separation of monosaccharides in the acid hydrolysates was performed on a CarboPac PA-1 analytical column (250 mm × 4 mm; Dionex Co.) at 25 °C. The neutral sugars were eluted with 18 mM NaOH, and the uronic acids were eluted with 100 mM NaOAc in 100 mM NaOH for 30 min at a constant flow rate of 1.0 mL/min. Sugar standards (arabinose, fucose, galactose, glucose, mannose, rhamnose, xylose, galacturonic acid, and glucuronic acid) were used to identify the sugars based on their retention times.

#### 3.3.3. Molecular Weight Determination

The molecular weight distributions of the polysaccharides were measured using a HPSEC system (JASCO PU-2089 Plus, Jasco, Tokyo, Japan) with a RI detector (JASCO RI-2031 Plus, Jasco, Tokyo, Japan). Asahipak GS-620, GS-520, and GS-320 columns (0.76 × 30 cm each; Showa Denko Co., Tokyo, Japan) were connected and used in the series. The column temperature maintained 40 °C, and the sample was eluted with 50 mm ammonium formate (pH 5.5 with formic acid) at a flow rate of 0.4 mL/min. A pullulan standard set (P-805, 336, 113, 48.8, 21.7, 10, 6, 1.32, 0.342 kDa; Sigma, St. Louis, MO, USA) was used to calibrate the standard curve, and the equation was as follows: log Mw = −0.0876 RT + 9.1538 (*R*^2^ = 0.9952).

#### 3.3.4. Infrared Analysis

The FT-IR spectra of polysaccharide samples were recorded using a Fourier-transform infrared spectrophotometer (FTIR-4600; Jasco, Tokyo, Japan). The samples were ground with KBr powder and then pressed into 1 mm pellets for FT-IR spectral measurement in the frequency range of 4000–400 cm^−1^.

### 3.4. Immunostimulatory Activities of Polysaccharides In Vitro

#### 3.4.1. Cell Culture

Murine RAW264.7 macrophages (Korean Cell Line Bank, Seoul, Korea) were cultured in Dulbecco’s modified Eagle medium containing 10% heat-inactivated fetal bovine serum (FBS) and 1% penicillin-streptomycin. The cells were incubated at 37 °C under 5% CO_2_.

#### 3.4.2. Cell Viability Assay

The effect of polysaccharides on the viability of RAW264.7 cells was assessed using a conventional cell counting kit-8 (CCK-8) (Cell Counting Kit, Dojindo, Tokyo, Japan) assay. Briefly, RAW264.7 cells (2 × 10^5^ cells/mL) were pre-cultured for 24 h and then stimulated with various concentrations of polysaccharides (1, 10, and 100 µg/mL) for 24 h. After the medium was removed, the cells were further incubated with 10 µL/well of CCK-8 solution for 90 min, and cytotoxicity was determined by measuring the absorbance at 450 nm.

#### 3.4.3. Phagocytic Activity Assay

The effects of polysaccharides on the phagocytic activity of macrophages were measured by neutral red phagocytosis assay. As described above, RAW264.7 cells (2 × 10^5^ cells/mL) were treated with the polysaccharide samples or LPS. Then, the culture medium was removed, and 100 µL/well of 0.1% neutral red was added and incubated for 30 min. After three washes with PBS, each of the wells was pipetted with 100 µL of cell-lysing solution (ethanol: 1 M acetic acid = 1:1), and the plate was statically placed for 1 h at room temperature. After the absorbance was measured at 540 nm, the phagocytic index was calculated using the following equation: Abs_sample_/Abs_blank control_.

#### 3.4.4. Measurement of NO and Cytokine Production

RAW264.7 macrophages (2 × 10^5^ cells/mL) were pre-incubated in 24-well plates for 24 h and then stimulated with the polysaccharide samples at 1, 10, and 100 µg/mL for 24 h. The culture supernatant was collected for the quantification of NO and cytokine production. Nitrite accumulation was measured using Griess reagent (Sigma, St. Louis, MO, USA), in which 100 μL of supernatant was mixed with 100 μL of Griess reagent solution and incubated for 15 min at room temperature. Next, the absorbance was measured at 540 nm. Meanwhile, the secretion of TNF-α, IL-6, and IL-12 from the macrophages were assessed using corresponding ELISA kits (BD Biosciences, Pharmingen, San Diego, CA, USA).

#### 3.4.5. Real-Time Quantitative PCR

RAW264.7 cells (4 × 10^5^ cells/mL) were pre-incubated in six-well plates for 24 h and then stimulated with polysaccharide samples for an additional 20 h. Subsequently, total RNA was extracted from stimulated RAW264.7 cells using NucleoSpin RNA (Macherey-Nagel, Duren, Germany), and 2 μg of RNA was reverse-transcribed into cDNA using ReverTra Ace qPCR RT Master Mix (Toyobo, Osaka, Japan). qRT-PCR was performed in a CFX96 Touch Real-Time PCR detection system (Bio-Rad, Hercules, CA, USA) with the SYBR Green Realtime PCR Master Mix (Toyobo, Osaka, Japan) in a 20 μL reaction volume. The oligonucleotide primers used for the PCR were as follows: inducible NO synthase (iNOS) (F: 5′-CCAGCCTGCCCCTTCAAT-3′, R: 5′-ATCCTTCGGCCCACCTTCCT-3′), TNF-α (F: 5′-AGGCACTCCCCCAAAAGATG-3′, R: 5′-CACCCCGAAGTTCAGTAGACAGA-3′), IL-6 (F: 5′-CCGGAGAGGAGACTTCACAGAG-3′, R: 5′-TCATTTCCACGATTTCCCAGAG-3′), IL-12 (F: 5′-CGTGCTCATGGCTGGTGCAAAG-3′, R: 5′-CTTCATCTGCAAGTTCTTGGGC-3′), GAPDH (F: 5′-CATGGCCTTCCGTGTTCCTAC-3′, R: 5′-TCAGTGGGCCCTCAGATGC-3′). The fold changes in gene expression levels were determined relative to the control GAPDH gene expression using the 2^−ΔΔ*C*t^ method.

### 3.5. Immuno-Enhancement Effects of ASEP-P In Vivo

#### 3.5.1. Experimental Animals

Specific pathogen-free KM male mice (seven–eight weeks of age, weighing 22 ± 2 g) were obtained from the Experimental Animal Center of the Yanbian University College of Medicine (Yanji, China). The mice were housed at 22 ± 2 °C and humidity of 40–60% under a 12 h light/dark cycle with free access to water and food. Our animal experiments were carried out under the protocols in accordance with the guidelines of the Medical Ethics Committee for the Use of Experimental Animals at Yanbian University (Medical Ethics Committee of Yanbian University Affliated Hospital #ID-0007, 20 March 2014).

#### 3.5.2. Animal Treatments

For the animal experiments with normal mice, the mice were randomly divided into four groups (*n* = 10 each) after acclimation to the experimental environment for one week. From day 1 to day 28, the groups were orally treated daily with saline (vehicle-treated control), ASEP-P (100 or 200 mg/kg/day), or CVT-E002™ (positive control: 100 mg/kg/day). CVT-E002™ (COLD-fX^®^; Afexa Life Sciences Inc., Edmonton, AB, Canada) is a commercially available herbal product for boosting the immune system, which primarily consists of poly-furanosyl-pyranosyl-saccharides (>80%) and oligosaccharides extracted using an aqueous method from the roots of *Panax quinquefolius* (North American ginseng) [26,35]. Twenty-four hours after the last administration, the mice were weighed and then sacrificed. The spleen and thymus indexes, and splenocyte proliferation were measured.

For the experiments with the immunosuppressed mice, the mice were randomly assigned to five groups (*n* = 10 each), and one group was used as a vehicle-treated control group, which did not receive any treatment to induce immunosuppression. From day 1 to day 3, the other four groups of mice were immunosuppressed using intraperitoneal injections of 100 mg/kg Cy, and one of the Cy-treated groups was used as an immunosuppressed model group. From day 4 to day 17, the five different groups of mice were orally administered daily as follows: vehicle-treated control (saline), Cy-induced immunosuppressed model (Cy and saline), and the ASEP-P groups (Cy and 100 or 200 mg/kg/day). CVT-E002™ (100 mg/kg/day) was also used as a positive control. Whole blood was collected for total WBC count 24 h after the last administration, and then the mice were sacrificed. The spleens were isolated for splenocyte proliferation and NK-cell activity analysis.

#### 3.5.3. Measurement of Organ Indexes

The spleens and thymuses were immediately removed and weighed 24 h after the last administration. The spleen index and thymus index were calculated according to the following formula: organ index = organ weight (mg)/mouse body weight (g). The spleen samples were also used to measure splenocyte proliferation.

#### 3.5.4. Splenocyte Proliferation Assay

The aseptically isolated spleens were washed with RPMI-1640 medium, gently ground, and then filtered through a 200-mesh steel sieve. Single-cell suspension was pooled in RPMI-1640 medium and then treated with lysis buffer (0.75% Tris–NH_4_Cl, pH 7.2) for five min in order to remove leukocytes. After centrifugation, the remaining cells were resuspended in RPMI-1640/FBS medium, and the number of cells per milliliter was counted. The splenocytes were placed in 96-well plates at 3 × 10^5^ cells/well containing concanavalin A (5 μg/mL) and LPS (10 μg/mL) for the T- and B-lymphocyte proliferation assays, respectively. After a 48 h culture, cell proliferation was measured by the CCK-8 assay, as described above in Section 3.4.2. Serum-free RPMI-1640 medium was used as the control.

#### 3.5.5. NK-Cell Activity Assay and WBC Counting

Splenic NK activity was assessed by an NK cell–mediated cytotoxicity assay in Yac-1 cells (NK-sensitive target cells). Briefly, splenocytes (effector cells) were added to the Yac-1 cells (1 × 10^5^ cells/well) in 96-well plates to obtain an effector-to-target cell ratio of 50:1 and then incubated for 4 h. The culture supernatants (100 μL) were mixed with 100 μL of lactate dehydrogenase solution (Promega, San Luis Obispo, CA, USA), and the absorbance was measured at 490 nm. From the blood samples collected, the WBC number was measured using a semi-automatic blood cell analyzer (KCTH04; Guangzhou Kingcare Medical Equipment Co., Ltd., Guangzhou, China).

### 3.6. Statistical Analysis

The data are presented as the means ± standard deviation. All statistical analyses were performed using the Statistical Package for the Social Sciences, version 20.0 (SPSS, Inc., Chicago, IL, USA). Statistical significance was determined by an unpaired *t*-test or one-way analysis of variance, followed by Duncan’s multiple range test; *p* < 0.05 was considered statistically significant.

## 4. Conclusions

In this study, polysaccharides from *Aster scaber* were successfully extracted using two different extraction methods: hot water extraction and enzyme-assisted extraction using amylase, cellulase, and pectinase. Their yields, physicochemical properties, and immunostimulatory activities were verified and compared. The results showed that all four polysaccharides (ASWP, ASEP-A, ASEP-C, and ASEP-P) were typical acidic hetero-polysaccharides, but their chemical contents and molecular weight distributions were considerably different depending on the extraction method. Moreover, the highest extraction yield and superior macrophage stimulatory effects of *Aster scaber* polysaccharides were obtained using pectinase assistance. It is well established that chemical and structural features, including monosaccharide composition, molecular weight, types of linkage, and side-chain distribution, are closely related to the immunostimulatory activity of specific polysaccharides [2]. Furthermore, the combinations of polysaccharides with different structural features also impact the resulting immunostimulatory activity [6]. Here, the high immunostimulatory activity of ASEP-P might be related to its higher content of KDO-like materials, rhamnose, and galactose, and to its different molecular weight profile, having more complex and various molecular weight values. In addition to inducing macrophage responses, immune-enhancing activities on normal and Cy-induced immunosuppressed mice were attributed to ASEP-P, thereby suggesting that ASEP-P can boost host immunity, irrespective of whether the host is in a normal or weakened state. From all the experimental results and analyses above, we concluded that the pectinase-assisted method was a better approach to extract immunostimulatory polysaccharides from *Aster scaber*, and ASEP-P has the potential to be a novel effective immune stimulator in functional food and medicinal products. However, the structural features and immunomodulatory mechanism of ASEP-P are still unclear, and thus further study on the structure-activity relationship is going on in our laboratory.

## Figures and Tables

**Table 1:** Yields and chemical properties of polysaccharides obtained from *Aster scaber* by different extraction methods. ASWP, a polysaccharide fraction obtained by hot water extraction; ASEP-A, a polysaccharide fraction obtained using an α-amylase-assisted extraction; ASEP-C, a polysaccharide fraction obtained using cellulase-assisted extraction; and ASEP-P, a polysaccharide fraction obtained using pectinase-assisted extraction from *Aster scaber*. Values are mean ± standard deviation, *n* = 3. Values followed by the different letter in the same row are significantly different (*p* < 0.05). ^(1)^ Percentage (%) in dried extract. ^(2)^ KDO, 2-keto-3-deoxy-mannooctanoic acid.

Samples	ASWP	ASEP-A	ASEP-C	ASEP-P
Yield (%)	3.3 ^ab^	3.4 ^ab^	3.1 ^b^	3.8 ^a^
Chemical composition (%) ^(1)^				
Neutral sugar	45.7 ^b^	59.9 ^a^	44.6 ^b^	58.5 ^a^
Uronic acid	51.6 ^a^	39.1 ^b^	54.1 ^a^	40.1 ^b^
Protein	2.3 ^a^	0.6 ^bc^	0.7 ^b^	0.3 ^c^
KDO ^(2)^-like material	0.4 ^b^	0.4 ^b^	0.6 ^b^	1.2 ^a^
Component sugar (molar ratio)				
Fucose	0.06	0.18	0.05	0.23
Rhamnose	0.44	1.59	0.62	2.08
Arabinose	1.00	0.75	1.25	1.11
Galactose	0.44	1.14	0.67	1.74
Glucose	1.00	1.00	1.00	1.00
Mannose	0.01	0.19	0.02	0.17
Xylose	0.03	0.13	0.05	0.14
Galacturonic acid	3.15	1.50	2.63	2.03
Glucuronic acid	0.05	0.13	0.04	0.18

**Table 2:** Effects of ASEP-P on body weight, immune organ indices, and lymphocyte proliferation in normal mice. ASEP-P (100 or 200 mg/kg) was administered daily for 28 days. CVT-E002™ (100 mg/kg) was used as a positive control (PC). The control group mice were treated with vehicle (normal saline solution). The data are expressed as the means ± standard deviation (*n* = 10). * *p* < 0.05, ** *p* < 0.01 compared with the vehicle-treated control group.

Group	Body Weight (g)		Immune Organ Indices (mg/g)		Splenocyte Proliferation (%)	
	Initial	Final	Spleen	Thymus	T-Lymphocytes	B-Lymphocytes
Control	22.00 ± 1.79	34.30 ± 3.46	0.62 ± 0.06	0.33 ± 0.13	100.0 ± 14.8	100.0 ± 11.8
ASEP-P (100 mg/kg)	22.13 ± 1.84	34.33 ± 3.28	0.70 ± 0.07	0.38 ± 0.09	113.1 ± 18.6 *	115.7 ± 19.4 *
ASEP-P (200 mg/kg)	21.75 ± 1.63	34.25 ± 3.93	0.74 ± 0.10 *	0.41 ± 0.10	125.1 ± 11.7 **	124.2 ± 20.2 **
PC (100 mg/kg)	21.94 ± 1.75	34.21 ± 2.93	0.76 ± 0.07 *	0.41 ± 0.13	124.4 ± 14.1 **	123.0 ± 13.8 **

## Supplementary Materials

Supplementary materials can be found at http://www.mdpi.com/1422-0067/19/9/2839/s1.

## Abbreviations

ASEP-A	amylase-assisted extracted polysaccharide
ASEP-P	pectinase-assisted extracted polysaccharide
ASEP-C	cellulase-assisted extracted polysaccharide
ASWP	hot water extracted polysaccharide
RG-II	rhamnogalacturonan-II
TNF-α	tumor necrosis factor-alpha
IL	interleukin
NO	nitric oxide
LPS	lipopolysaccharide
PBS	phosphate-buffered saline
ELISA	enzyme-linked immunosorbent assay
qRT-PCR	quantitative reverse transcription–polymerase chain reaction
GAPDH	glyceraldehyde-3-phosphate dehydrogenase
KM	Kunming
NK	natural killer
WBC	white blood cell
KDO	2-keto-3-deoxy-mannooctanoic acid
HPSEC	high-performance size-exclusion chromatography
RI	refractive index
FT-IR	fourier-transform infrared spectroscopy
