# Supplementary figures and images for: The Effect of Pectinase-Assisted Extraction on the Physicochemical and Biological Properties of Polysaccharides from Aster scaber
Source: Int J Mol Sci. 2018 Sep 19;19(9):2839. doi: 10.3390/ijms19092839 (PMC6164288; doi:10.3390/ijms19092839)

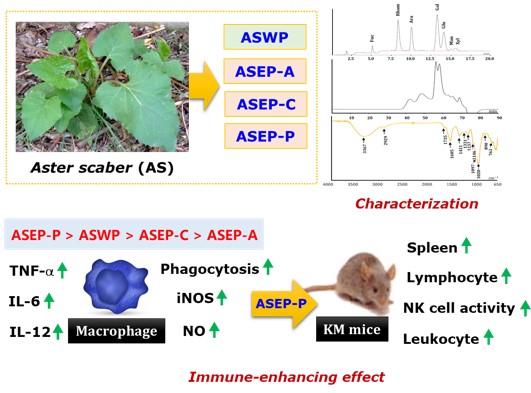

Supplement: Supplementary file 1 [file ijms-19-02839-s001.jpg]
